# TLR4 signalling in ischemia/reperfusion injury: a promising target for linking inflammation, oxidative stress and programmed cell death to improve organ transplantation outcomes

**DOI:** 10.3389/fimmu.2024.1447060

**Published:** 2024-07-18

**Authors:** Sutian Wang, Kunli Zhang, Qiuyan Huang, Fanming Meng, Shoulong Deng

**Affiliations:** ^1^ State Key Laboratory of Swine and Poultry Breeding Industry, Guangdong Key Laboratory of Animal Breeding and Nutrition, Institute of Animal Science, Guangdong Academy of Agricultural Sciences, Guangzhou, China; ^2^ Institute of Animal Health, Guangdong Academy of Agricultural Sciences, Key Laboratory of Livestock Disease Prevention of Guangdong Province, Scientific Observation and Experiment Station of Veterinary Drugs and Diagnostic Techniques of Guangdong Province, Ministry of Agriculture and Rural Affairs, Guangzhou, China; ^3^ Institute of Laboratory Animal Sciences, Chinese Academy of Medical Sciences and Comparative Medicine Center, Peking Union Medical College, Beijing, China

**Keywords:** toll-like receptor 4, ischemia/reperfusion injury, organ transplantation, inflammation, oxidative stress, programmed cell death

## Abstract

Transplantations represent the principal therapeutic interventions for terminal organ failure, a procedure that has salvaged myriad lives annually. Ischemia/reperfusion injury (IRI) is frequently correlated with an unfavourable prognosis and is relevant for early graft dysfunction and graft survival. IRI constitutes a complex pathological state influenced by a series of factors such as oxidative stress, metabolic stress, leukocytic infiltration, programmed cell death pathways, and inflammatory immune responses. Reducing ischemia/reperfusion injury is one of the main directions of transplantation research. Toll-like receptors (TLRs) are important pattern-recognition receptors expressed on various organs that orchestrate the immune responses upon recognising PAMPs and DAMPs. Targeting the TLR4 signalling has recently been suggested as a promising approach for alleviating IRI by affecting inflammation, oxidative stress and programmed cell death (PCD). In this minireview, we summarise the role of TLR4 signalling in regulating inflammation, oxidative stress and PCD in organ transplantation and discuss their interactions during IRI. A detailed understanding of the multiple functions of TLR4 in IRI provides novel insights into developing therapies to improve organ transplantation outcomes.

## 1 Introduction

Toll-like receptors (TLRs) serve as important pathogen recognition receptors that efficiently coordinate the innate immune response, subsequently facilitating the adaptive immune response. TLRs can be activated by exogenous ligands expressed by invading pathogens and interact with endogenous ligands released during cellular damage. For years, studies and statistics have shown that TLRs are pivotal contributors to the multifaceted aspects of transplantation biology across diverse organs, including ischemia/reperfusion injury (IRI), graft rejection and tolerance, and post-transplantation infections (1). TLR4 is a member of the TLR family and mainly expresses the cytoplasmic membrane of various cells, such as macrophages, monocytes and dendritic cells (2). A large amount of TLR4 is also detected in the kidney, liver, heart, intestine and brain (3). TLR4 is activated by specific pathogen-related molecular patterns (PAMPs) (such as bacterial LPS) and endogenous damage-associated molecular patterns (DAMPs) (such as heat shock protein and high-mobility group box protein-1). Activation of TLR4 by PAMPs or DAMPs would initiate the innate and adaptive immune responses via inflammatory cytokines, chemokines and cell surface molecules (4). TLR4 activation also relates to effector functions such as phagocytosis, REDOX balance and programmed cell death (PCD) (5–7). Mutations in the TLR4 gene affect these processes (8).

As the most important treatment for organ failure, organ transplantation is one of the most promising ways to rescue patients with clinical emergencies. However, in organ acquisition and transplantation processes, IRI is relevant for early graft dysfunction and survival, which aggravates graft damage. A compromised blood flow inevitably induces hypoxia, which further leads to redox imbalance of the donor graft. Reperfusion of blood induces oxidative injury and elicits an inflammatory cascade upon reoxygenation, exacerbating allograft impairment and dysfunction (9). In addition, this series of events that occur during IRI activates the innate immune and PCD (10). However, the interaction between them remains unclear. Studying these issues will help maintain the function of the graft. Here, we summarise the multifunctional roles of TLR4 signalling in IRI from the perspective of innate immunity. Moreover, we discuss the crosstalk between TLR4, inflammation, oxidative stress and PCD to provide a promising reference for treating IRI following graft transplantation.

## 2 TLR4 signalling in ischemia/reperfusion injury

TLR4 is known for its ability to recognise a diverse array of substances, including bacterial lipopolysaccharide (LPS), viral structural proteins and fungal mannan, thereby playing a pivotal role in activating innate immunity. For instance, upon infection by Gram-negative bacteria, TLR4 recognizes LPS through LPS binding protein, myeloid differentiation factor 2 (MD-2) and CD14. Then TLR4 undergoes oligomerisation and orchestrates the recruitment of downstream adaptor proteins via its Toll-interleukin-1 receptor domains, thereby initiating signal transduction cascades. Like other TLRs, TLR4 engages in two distinct modes of signal transduction. Upon association with MyD88, phosphorylated IRAK-4 triggers the activation of the TRAF6-TAK1-NF-κB/MAPK signalling axis, culminating in the secretion of inflammatory cytokines, such as IL-1β, IL-6, and TNF-α. These cytokines mediate inflammation, nitroxidative stress and PCD (7). In the MyD88-independent pathways, TRAM is selectively recruited to TLR4 to facilitate the linkage between TLR4 and TRIF, thereby inducing the production of type I interferons and other proinflammatory cytokines through the activation of IRF3, nuclear factor kappa B (NF-κB), and mitogen-activated protein kinases (MAPK) pathways.

Although most research on TLR4 has focused on its role in pathogenic microbial infections, the effect of TLR4 on IRI is of concern. Because TLR4 is highly expressed in many tissues and organs and can induce immune response, its influence on the IRI in various organ grafts has been studied. There are consistent reports on the role of TLR4 in IRI: TLR4 activation aggravates IRI, while TLR4 deficiency has a protective effect on IRI. Studies have shown that the expression of TLR4 was increased in heart failure and ischemic heart (11). Further research has demonstrated the TLR4-MyD88 pathway participated in myocardial IRI. TLR4 deficiency, TLR4 antagonist or MyD88 deficiency significantly reduced myocardial IRI, decreased myocardial infarction and improved cardiac function after myocardial IRI. A similar research also found that TLR4^(−/−)^ mice could protect themselves from renal I/R. Moreover, TLR4 deficiency improved renal function, chemokine production and cellular infiltration after renal IRI (12). Knocking out molecules downstream of TLR4 signallings, such as MyD88 or TRIF, significantly ameliorates chronic allograft damage and fibrosis after renal transplantation. Also, TLR4 deficiency ameliorated hepatic injury after liver ischemia through TNF-α inhibition. Meanwhile, TLR4 gene deficiency attenuated ischemia-induced brain damage and reduced brain infarct size through PI3K/Akt signalling. An interesting study showed that TLR4 Deficiency attenuated lung damage after intestinal I/R by inhibiting cell death and production of proinflammatory cytokines (13). TLR4-NF-κB/p38 MAPK pathways play important roles in intestinal I/R-induced acute lung injury. Although it is widely believed that inhibiting TLR4 can improve the outcome of transplant recipients, the specific mechanism of TLR4 signalling in this process is still under investigation.

## 3 Crosstalk between TLR4 signalling, inflammation, oxidative stress and PCD in IRI

### 3.1 TLR4: a core node that interacts with inflammation, oxidative stress and PCD in IRI

IRI is divided into warm IRI and cold IRI. Warm IRI mostly occurs during organectomy, while cold IRI mostly occurs during cardiac death organ donation transplantation. The distinctive features of IRI include the generation of reactive oxygen species (ROS), cytokine production, complement system activation, eicosanoid synthesis, infiltration of activated leukocytes, mitochondrial impairment, and a combination of necrosis, apoptosis autophagy and pyroptosis. During reperfusion, DAMPs released from apoptotic and necrotic cells stimulate Kupffer cells to produce inflammatory mediators, such as chemokines, cytokines and reactive oxygen species, to stimulate the next stage of reperfusion injury by coordinating homing of neutrophils (14). In addition, studies have shown that the damage caused by warm ischemia is more serious than that caused by cold ischemia. Warm ischemia is usually harmful to cellular and molecular pathways, and the generation of ROS induced by warm ischemia and the release of many inflammatory mediators aggravate the damage caused by early reperfusion. IRI caused by warm or cold ischemia is closely related to inflammation, redox damage and PCD, which are the main pathophysiological mechanisms of IRI.

**Figure 1:**
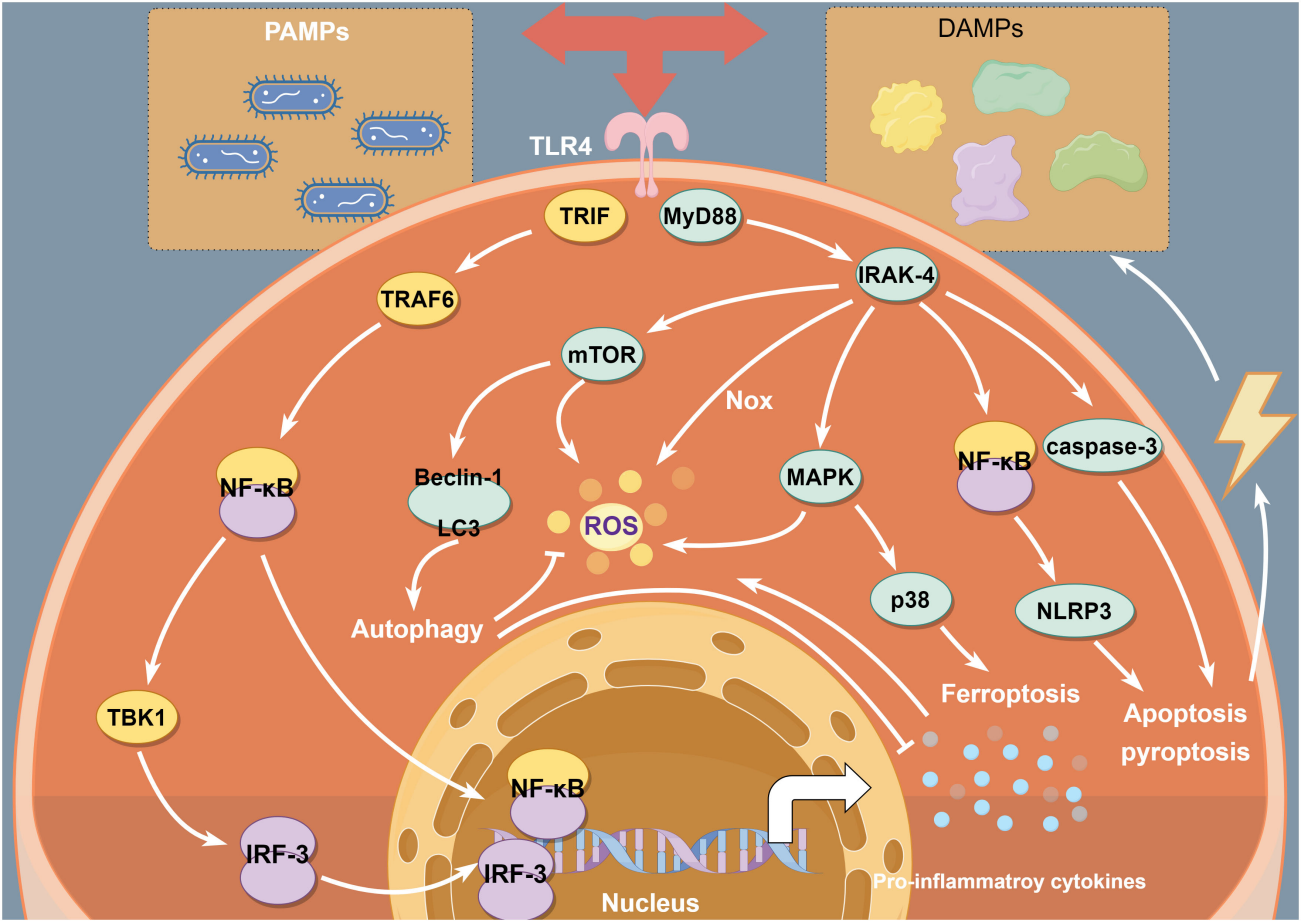
TLR4 interacts with inflammation, oxidative stress and PCD in IRI. TLR4 signalling induces inflammatory response mainly via TLR4-NF-κB signal. Moreover, MAPKs/mTOR/Nox pathways are involved in ROS generation. In addition, TLR4- NF-κB/MAPK/mTOR signalling pathways contribute to triggering PCD, which have important impact on intracellular inflammation and redox homeostasis during IRI.

TLR4 is expressed in all kinds of tissue and organs and plays key roles in the aseptic inflammatory cascade of IRI. IRI leads to aseptic inflammation, activation of inflammatory cells, production of many oxidising intermediates, and induces PCD, which can damage parenchymal cells and release DAMPs. TLR4 can recognise and bind to DAMPs and trigger inflammatory cascades through the proinflammatory cytokines and chemokines produced downstream of TLR4, leading to aggregation of neutrophils, T lymphocytes and organ dysfunction (15). This process accompanies the production of large amounts of proinflammatory cytokines, further exacerbating IRI. The TLR4 signalling plays an important role in the pathogenesis of IRI and is also the main pathway leading to cytokine release. Furthermore, recent studies have found that TLR4 also has a complex interaction with oxidative stress and PCD in IRI (**Figure 1**). Thus, the understanding of the TLR4 function needs to go further.

### 3.2 TLR4 signalling and inflammatory responses in IRI

It has been fully explored that the inflammation of post-transplant IRI was induced by TLR4 signalling. The transplant surgery inevitably results in tissue injury, oxidative dysregulation, or bacterial translocation, which serve as PAMPs and DAMPs to activate inflammatory cells. On the one hand, under the influence of surgical incisions and intestinal stress, clinical hepatic and intestinal transplantation procedures trigger the translocation of commensal microflora, which further induces innate immune responses through the activation of TLRs. On the other hand, IRI can also trigger the upregulation of various DAMPs, including high-mobility group box chromosomal protein 1 (HMGB1) and heat shock protein (HSP), fibrinogen, hyaluronan, and biglycan. Each of these molecules can be recognised by TLR4 and activate the innate immune response. HMGB1 serves as a crucial chromosomal binding protein, mainly distributed within the cell nuclei of eukaryotes, playing essential roles in the lived body. In the usual state, HMGB1 predominantly resides within the nuclei. Upon exposure to specific external stimuli, such as ischemic conditions and pathogenic invasion, HMGB1 undergoes acetylation, translocates to the cytoplasm, and is actively secreted by inflammatory cells or passively released by necrotic cells. Consequently, it functions as a “necrosis marker” recognised by the innate immune receptor, contributing to the initiation and progression of inflammatory responses in diverse pathological conditions, including IRI. Extracellular HMGB1 exerts its effects through interactions with TLR4. Many studies have demonstrated that HMGB1 can activate NF-κB and MAPK pathways and further mediate inflammatory responses through TLR4. The impact of HMGB1 in IRI primarily hinges on the expression of the TLR4 receptor on macrophages. HMGB1-TLR4 pathway triggers the generation of proinflammatory cytokines through the activation of MAPKs and downstream NF-κB signalling in the context of IRI. During IRI, the damaged epithelial and endothelial cells secrete HMGB1. After renal injury, the interaction between TLR4 receptors and HMGB1 promote IL-6 secretion by the injured renal cells. In a recent study, during renal IRI, the HMGB1-TLR4 pathway promotes IL-23 and IL-17A productions, which activate macrophage and mediate the recruitment and migration of neutrophils (16). HSP is another class of DAMPs that mediates TLR4-dependent release of inflammatory cytokines. In an animal liver transplantation model, TLR4 and HSP60/70 expression significantly increased after reperfusion (17). HSP60 can induce IRAK-1 activation through TLR4-MyD88 pathways and then initiate inflammatory responses. TLR4 deletion or anti-HSP60 antibody attenuates I/R-induced cytokine expression, including IL-1β and IL-6. However, the role of HSP70 in IRI remains controversial. On the one hand, HSP70 can initiate inflammatory pathways downstream of TLR4 and damage the organ. On the other hand, overexpression of HSP70 has demonstrated protective effects against renal injury by activating rapid repair and survival mechanisms via Akt signalling (18). In addition, HSP32 ameliorates liver inflammatory cell infiltration by inhibiting TLR4 –MyD88 and TLR4-TRIF pathways, decreasing phosphorylation of IRF3, p38, IκB-α and NF-κB and up-regulating negative regulators of TLR4 signalling. Studies show that fibrinogen, hyaluronan, and biglycan significantly increase after IRI. A recent research evaluated the pathogenetic role of hyaluronan in Intestinal IRI (19). Elevated hyaluronan level after intestinal IRI promotes the proliferation of pathogenic bacteria, increases neutrophil infiltration, up-regulates the expression of TLR4, and induces a pro-inflammatory state. Studies on TLR4 mediating inflammatory responses in IRI will continue. Knowing more about the mechanisms involved could help us develop more effective treatments.

### 3.3 TLR4 signalling and oxidative stress in IRI

Ischemia-reperfusion injury is a phenomenon characterised by the exacerbation of damage to a hypoxic organ upon the return of oxygen supply. Oxidative stress is one of the key factors in the IRI mechanism. Studies have confirmed that ischemia-reperfusion injury can lead to the production of ROS, and ROS can correspondingly cause organ damage and dysfunction. In the stage of ischemia and hypoxia, tissues and organs may undergo pathological changes. The decrease in ATP synthesis and increased metabolic decomposition will stimulate ROS production. Due to the insufficient ability of the body to clear ROS during hypoxia, the accumulated reactive oxygen species will react with organic macromolecules and other components, thus leading to oxidative damage. In some cases, reperfusion will aggravate the damage and pathological changes between blood and ischemic tissue. The reperfusion reoxygenation stage will produce a large number of free radicals. The ROS produced in the early stage can directly cause cell damage (even cell death) or indirectly cause damage by inducing the activation of inflammatory responses, eventually leading to aggravation of injury (20).

A series of studies have confirmed that ROS production in IRI includes the following pathways: mitochondrial pathway, xanthine oxidase pathway, neutrophils pathway, catecholamines oxidation pathway and calcium overload. Few studies have focused on the interaction between TLR4 and oxidative stress in IRI. In IRI-induced cerebral injury, Wang et al. found the activated TLR4- NF-κB pathways could induce more oxygen radicals via aggravating inflammatory responses (21). TLR4 nonfunctional mutation mice are shielded from hepatic warm IRI, exhibiting diminished levels of ROS, cytokines, and HMGB1 (22). It is known to all that TLR4-MyD88 signalling can activate TRAF6. TRAF6 facilitates mitochondrial ROS generation by directly ubiquitylating domains of evolutionarily conserved signalling intermediates in Toll pathways (ECSIT), a pivotal factor in mitochondrial respiratory chain assembly (7). Moreover, TLR4 can interact directly with NADPH oxidase (NOX). Actually, the TLR4 TIR domain interacts directly with the carboxy-terminal region of Nox2/4. The activation of NADPH oxidase triggers the transmembrane electron transport and ROS generation. TLR4 facilitates the upregulation of inducible nitric oxide synthase (iNOS) through NF-κB signalling, leading to nitric oxide (NO) generation that inhibits superoxide dismutase (SOD) function and enhances malondialdehyde (MDA) production, exacerbating nitroxidative stress. In addition, TLR4-mediated proinflammatory cytokines, such as TNF-α and IL-1β, also stimulate ROS production (23). The mammalian target of rapamycin (mTOR) is a key factor in ROS generation. TLR4 can affect mTOR activity via MAPK or PI3K signalling. So TLR4-MAPK-mTOR and TLR4-PI3K-mTOR pathways should also be of concern in inducing oxidative stress. Interestingly, ROS is involved in mediating TLR4-NF-κB/MAPK pathways, which can further induce the production of proinflammatory cytokines. TLR4 activation and oxidative stress may be reciprocal causations and aggravate IRI. In each stage of IRI, the mechanism of mutual activation and regulation between them still needs further study.

### 3.4 TLR4 signalling and programmed cell death in IRI

**Figure 2:**
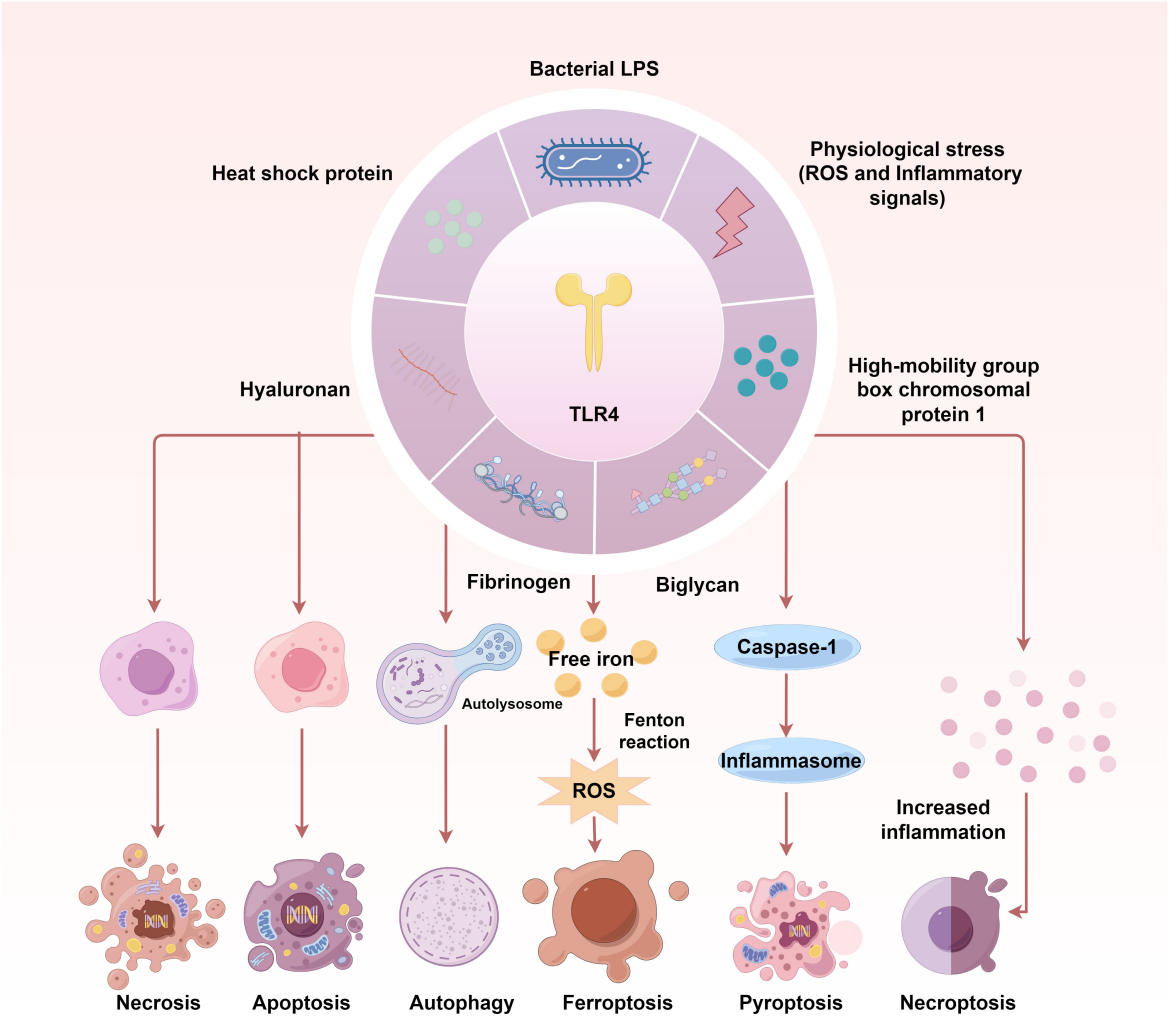
The TLR4 signalling contributes to multiple programmed cell death pathways in IRI. TLR4 activation and its downstream signal transduction are key to initiating multiple PCD during IRI. The TLR4- NF-κB-NLRP3 pathway induces apoptosis and pyroptosis via Bcl-2/Bax/Caspase-3 axis in IRI. TLR4-MyD88-MAPK/NF-κB and TLR4/PI3K/Akt/mTOR signalling cascades contribute to IRI-induced autophagy. In turn, autophagy can affect the activation of TLR4, inflammatory responses and redox levels. DAMPs-triggered ferroptosis can be attributed to the decreasing ATP, Fe2+ and transferrin 1 protein levels and increasing ferritin 1 expression during IRI, which are regulated by TLR4-p38MAPK and TLR4-NOX4 signals. TLR4-NOX4-mediated ROS is one of the most important factors triggering ferroptosis. The TLR4-RIPK3-FUNDC1 axis has been demonstrated to activate necroptosis.

The TLR4 signalling contributes to multiple PCD pathways, including apoptosis, necroptosis, autophagy, pyroptosis and ferroptosis (**Figure 2**). During IRI post-transplantation, various PCD occur together. TLR4 signalling is crucial for gaining a deeper understanding of their relation.

TLR4 signal transduction is highly correlated with apoptosis (24, 25). TLR4- NF-κB pathway activates the transcription of NOD-like receptor pyrin domain-containing protein 3 (NLRP3) components. Then, NLRP3 generates a complex with the apoptosis-associated speck-like protein containing caspase-recruitment domain, which can associate with the cysteine protease caspase-1 to form inflammasomes. This process can trigger caspase-1 activation, influencing the Bcl-2/Bax/Caspase-3 axis and apoptotic body formation, thereby potentially enhancing inflammation, apoptosis, and inflammation-associated pyroptosis. Some other studies showed TLR4-MAPK and TLR4-Akt-mTOR signalling pathways were also involved in IRI-induced apoptosis (26, 27). TLR4 antagonists and inhibitors have ameliorated apoptosis in IRI and transplant models. Moreover, a series of miRNAs can alleviate apoptosis of parenchymal cells via mediating TLR4 signalling after IRI. For instance, miR-27a interacts with 3’ UTR of TLR4 to down-regulate TLR4 expression and inhibits inflammation, cell adhesion and apoptosis in renal IRI (28). Other antagonists, inhibitors or miRNAs that target upstream or downstream molecules of TLR4, such as panaxynol for TLR4-NF-κB pathway in cardiac IRI, clemastine fumarate for TLR/Akt pathway in pathway in myocardial IRI, miR-143–3p for TLR4-NF-κB pathway in myocardial IRI, can effectively relieve the IRI by inhibiting inflammation and apoptosis (29–31). The development and utilisation of these molecules are the focus of current research. However, the more detailed mechanism has yet to be resolved.

The involvement of autophagy in regulating IRI has attracted much attention in recent years. The common idea is that autophagy alleviates IRI following transplantation (32). TLR4 can modulate autophagy through the TLR4-MyD88-MAPK/NF-κB and TLR4/PI3K/Akt/mTOR signalling cascades (7). Berberine, a well-known quaternary isoquinoline alkaloid, targets the HMGB1/TLR4/NF-κB pathway to inhibit inflammatory responses and oxidative stress and contributes to inducing autophagy by up-regulating Beclin-1 and LC3-II (33). However, another study showed that inhibiting TLR4 signalling and autophagy activation by Dunaliella salina, a halophilic unicellular microalga, can mediate anti-inflammatory and anti-apoptotic activities and antagonise myocardial IRI. In addition, as a high-affinity ligand of TLR4, biglycan can mediate TLR4 signal transduction. Soluble biglycan triggers autophagy by evoking autophagic flux (34). Interestingly, biglycan induces CD14/TLR4-dependent proinflammatory M1 macrophage recruitment and tissue injury while concurrently promoting CD44/TLR4-dependent M1 macrophage autophagy activation, ultimately facilitating M2 macrophage polarisation and tissue repair in subsequent phases. The state of balance between these two pathways will determine the pathological outcome. It is widely believed that autophagy is a “double-edged sword” (35). Moderate autophagy maintains physiological homeostasis, but excessive autophagy aggravates physiological damage. These different results may be attributed to different stimulation times/intensities, different tissues and organs, and different signal transduction pathways. Hence, more investigations are needed to analyse the specific mechanism under certain pathological circumstances and ascertain its universality to other pathogenic processes.

Ferroptosis is a recently recognised form of myocardial cell death characterised by iron-dependent lipid peroxidation, primarily triggered by redox imbalance. Ferroptosis participates in pro-inflammatory cytokine production during IRI following transplantation. Generally, ferroptosis probably enhances various DAMPs generation and alarmins that activate all kinds of cells within different tissues and organs (36). Endogenous molecules released during ferroptosis activate TLR4 signalling in endothelial cells. In turns, the excessive activation of TLR4 can trigger redox imbalance and severe lipid peroxidation, which are the major causes of ferroptosis (37). Recently, a growing body of research has found a close relationship between TLR4 signalling and ferroptosis. Oxysophoridine can inhibit the TLR4-p38MAPK pathway, further decreasing ATP, Fe^2+^ and transferrin 1 protein levels, increasing ferritin 1 expression, and finally alleviating ferroptosis (38). Lysine-specific demethylase 1 exacerbates oxidative stress and ferroptosis triggered by renal ischemia and reperfusion injury via TLR4-NOX4 signalling (39). TLR4-NOX4 pathway closely links to the production of oxidising intermediate. Puerarin mitigated oxidative stress and ferroptosis during IRI-induced renal fibrosis through TLR4-NOX4 signalling (40). Necroptosis and pyroptosis are associated with cellular inflammation (41, 42). During IRI, the TLR4-NF-κB-NLRP3 signal axis is identified as the predominant pathway for pyroptosis, and the TLR4-RIPK3-FUNDC1 axis has been demonstrated to activate necroptosis. Notably, as a stress-response protein, cold-inducible RNA-binding protein (CIRP) can induce necroptosis and pyroptosis via simultaneously activating NF-κB. Anti-CIRP Ab or CIRP-derived small peptides may have effective therapeutic potentials in organ IRI. Currently, most of the research is still preliminary and more precise studies are required to elucidate the crosstalking between TLR4 and PCD (43).

## 4 Conclusions and prospects

The factors that affect the outcome of organ transplantation are complex, and the role of innate immunity in this process is of concern. Cell receptors can detect mechanical injury and graft IRI, triggering inflammatory responses, oxidative stress, PCD, and adaptive immunity. The TLR4 signalling is a crucial target for alleviating IRI after transplantation. Most previous research has only focused on TLR4’s function in one of these areas. IRI is not only the result of multiple factors but also the source of many responses, including inflammation, oxidative stress and PCD. At the same time, TLR4 is an important node connecting inflammation, oxidative damage and PCD. Under different conditions, the results of their interactions vary greatly, resulting in different prognoses. The next step should be to analyse their relationship more comprehensively and seek more evidence to clarify their mechanisms.

## Author contributions

SW: Writing – review & editing, Writing – original draft, Funding acquisition, Conceptualization. KZ: Writing – review & editing, Writing – original draft, Conceptualization. QH: Writing – review & editing. FM: Writing – review & editing. SD: Writing – review & editing, Writing – original draft, Conceptualization.

## Glossary

**Table:**

TLRs	toll-like receptors
IRI	Ischemia/reperfusion injury
PAMP	pathogen-related molecular pattern
DAMP	damage-associated molecular pattern
PCD	programmed cell death
LPS	lipopolysaccharide
MD-2	myeloid differentiation factor 2
NF-κB	nuclear factor kappa B
MAPK	mitogen-activated protein kinase
ROS	mitogen-activated protein kinase
HMGB1	high-mobility group box chromosomal protein 1
HSP	heat shock protein
ECSIT	evolutionarily conserved signalling intermediates in Toll pathway
NOX	NADPH oxidase
iNOS	inducible nitric oxide synthase
NO	nitric oxide
SOD	superoxide dismutase
MDA	malondialdehyde
mTOR	mammalian target of rapamycin
NLRP3	NOD-like receptor family pyrin domain containing 3
CIRP	cold-inducible RNA-binding protein
